# Post translational changes to α-synuclein control iron and dopamine trafficking; a concept for neuron vulnerability in Parkinson’s disease

**DOI:** 10.1186/s13024-017-0186-8

**Published:** 2017-06-07

**Authors:** James A. Duce, Bruce X. Wong, Hannah Durham, Jean-Christophe Devedjian, David P. Smith, David Devos

**Affiliations:** 1 School of Biomedical Sciences, Faculty of Biological Sciences, University of Leeds, Leeds, West Yorkshire UK; 2 Oxidation Biology Unit, the Florey Institute of Neuroscience and Mental Health, the University of Melbourne, Parkville, VIC Australia; 3 Department of Medical Pharmacology, Lille University, INSERM U1171, CHU of Lille, Lille, France; 4 Biomolecular Research Centre, Sheffield Hallam University, Howard Street, Sheffield, UK

**Keywords:** α-synuclein, Iron, Dopamine, Endosomal trafficking, Oxidative stress, Post translational modification, N-terminal acetylation, Phosphorylation, Oxidation

## Abstract

Parkinson’s disease is a multifactorial neurodegenerative disorder, the aetiology of which remains elusive. The primary clinical feature of progressively impaired motor control is caused by a loss of midbrain substantia nigra dopamine neurons that have a high α-synuclein (α-syn) and iron content. α-Syn is a neuronal protein that is highly modified post-translationally and central to the Lewy body neuropathology of the disease. This review provides an overview of findings on the role post translational modifications to α-syn have in membrane binding and intracellular vesicle trafficking. Furthermore, we propose a concept in which acetylation and phosphorylation of α-syn modulate endocytic import of iron and vesicle transport of dopamine during normal physiology. Disregulated phosphorylation and oxidation of α-syn mediate iron and dopamine dependent oxidative stress through impaired cellular location and increase propensity for α-syn aggregation. The proposition highlights a connection between α-syn, iron and dopamine, three pathological components associated with disease progression in sporadic Parkinson’s disease.

## Background

Parkinson’s disease (PD) is the second most common progressive neurodegenerative disorder and is characterised by tremor, bradykinesia rigidity and gait disorders [1]. Additive non-motor symptoms include cognitive deficits, sleep disturbances, anxiety, motivation disorders and mood disorders [2]. The pathophysiology of PD is characterised by loss of over 60% of neuromelanin-containing dopaminergic neurons in the substantia nigra pars compacta (SNc), which results in a > 90% reduction in dopamine (DA) in the striatum causing the motor symptoms observed with the disease. PD is also neuropathologically characterised by the presence of Lewy bodies (LBs) and Lewy neurites (LN). These cytoplasmic inclusions contain α-synuclein (α-syn; PARK1) as the major constituent [3]. As well as in the SNc, LBs are found in brain regions including the locus coeruleus, raphe nucleus and dorsal motor nucleus of the vagus [4]. These pathological hallmarks are also observed in several other synucleinopathies, including multiple system atrophy (MSA) and dementia with Lewy bodies (DLB), as well as some forms of neurodegeneration with brain iron accumulation (NBIA) [5].

An understanding of why SNc neurons are vulnerable in familial forms of PD or with advancing age in specific environments may yield important insights into the disease process and avenues into ways in which to therapeutically intervene. There have been several theories as to the susceptibility of dopaminergic neurons in the SNc in PD, in particular, the Ca^2+^-dependent pacemaking capability of these neurons that leads to a maintained elevated mitochondrial oxidant stress (OS) [6]. However, this review attempts to identify a pathogenic mechanism of relevance to PD when DA, iron and α-syn are highly expressed within the same neuron. We propose a possible association between post-translational modifications to α-syn, altered neurotransmitter compartmentalisation at the synapse and enhanced iron-dependent OS.

Before the concept can be presented, an overview of prior relevant literature must be appraised. The current comprehension on DA and iron metabolism, as well as the role α-syn has in synaptic maintenance, are provided before evaluating the genetic and post-translational modifications that reportedly alter α-syn structure and function.

## Oxidative stress and dopamine metabolism

The majority of midbrain dopaminergic neurons are located in the SNc and ventral tegmental area (VTA) and have widespread projections [7]. DA is a monoaminergic neurotransmitter produced from tyrosine in the cytosol as a two-step reaction (for further information refer to [8]). Iron acts as a co-factor for the tyrosine hydroxylase (TH) step in the catalysis of DA synthesis [9].

Within certain environments, such as in the presence of divalent metals, the unstable catechol ring of DA can be enzymatically deaminated by monoamine oxidase (MAO). This generates dihydroxyphenylacetic acid (DOPAC) and hydrogen peroxide (H_2_O_2_) as well as subsequent oxidative species and hydroxyl radicals [10]. Alternatively, DA can be spontaneously oxidised in the presence of oxygen to yield DA-*o*-quinone, H_2_O_2_ and superoxide [11]. The end product aminochrome can also participate in oxidative stress and mitochondrial dysfunction [12] as well as induce and stabilise neurotoxic oligomeric α-syn formation [13].

In the cytoplasm, DA is highly prone to spontaneous and enzymatic degradation. To minimise the risk in exposing this monoaminergic neurotransmitter to an oxidative environment, the SNc neuron compartmentalises DA in vesicles. By virtue of their low pH and MAO-free environment, storage in synaptic vesicles hinders DA breakdown. Immediately after cytosolic synthesis, DA is taken up into synaptic vesicles by the vesicular monoamine transporter 2 (VMAT2). DA released from the synapse that is reinternalised into the nerve terminal through dopamine transporter (DAT) is also repackaged in synaptic vesicles via VMAT2. Several studies have shown that intracellular accumulation of DA or the modification of these DA transporters can lead to neurotoxicity [14, 15].

## Alteration in iron metabolism

Physiological iron is important for the regulation of cell development, mitochondrial respiration, production of myelin [16] as well as neurotransmitter synthesis and metabolism [17]. Iron is of particular importance and abundance in the SNc [18], in part due to its requirement as a cofactor for TH activity which is vital for DA synthesis. It is the ability of iron to transition in valency between ferrous (Fe^2+^) and ferric (Fe^3+^) that makes it an essential element for cell survival. However when this electron transfer is not correctly harnessed within an aerobic environment the resulting Fenton reaction produces hydroxyl radicals that induce OS toxicity [19, 20]. Iron deficiency can lead to cognitive impairment [21, 22] but excess iron is also an underlying factor associated with neuropathology in neurodegenerative disorders such as PD [17, 19, 23]. As such it is of upmost importance that iron levels and redox state are carefully regulated in the brain so as to maintain optimal neuronal function while avoiding toxicity.

Transferrin is a glycoprotein that binds and transports iron throughout the extracellular system including in the brain [24]. When required, cellular uptake of iron within holo-transferrin (transferrin with iron attached) predominantly occurs via transferrin receptor (TfR) mediated endocytosis [17]. Despite TfR mediated internalisation being the predominant pathway for iron import in neurons, other known import mechanisms include divalent metal transporter 1 (DMT1). Once inside the cell the cytosolic iron can be utilised for the functional requirements of maintaining a healthy cell, safely stored or exported from the cell. Storage is typically within ferritin but select cell types, including dopaminergic neurons of the SNc, alternatively store iron in neuromelanin. These neuromelanin positive cells tend to express ferritin poorly [25, 26]. When the cell has sufficient iron to maintain survival, excess is removed through the only known membrane pore protein ferroportin (FPN). For iron efflux, FPN must be maintained on the cell surface and stabilisation is assisted in neurons by the type 1 transmembrane protein amyloid-β precursor protein (APP) [27, 28]. In contrast, FPN stabilisation on astrocytes is through ceruloplasmin and oligodendrocytes use hephaestin [29, 30]. Expression of iron regulating proteins is controlled through the canonical cis-trans iron regulatory system involving iron response protein binding to an iron response element (IRE) (for review see [31]). However an important aspect also to consider when investigating the iron regulatory function of these proteins is their location within the cell, as illustrated with the required membrane location of FPN.

The susceptibility of SNc neurons in PD is in part considered to be due to their iron content [32]. A decrease in ferritin that parallels the reduced iron [33] suggests intracellular iron may be more available for reactive oxygen species (ROS) generation by Fenton reactions [34, 35]. Changes to various other proteins involved in iron homeostasis have also been observed in PD. Altered DMT1 expression correlates with iron accumulation in the SNc ventral tier of a Parkinsonian toxicity mouse model (1-methyl-4-phenyl-1,2,3,6-tetrahydropyridine; MPTP) as well as PD patients [36, 37]. In contrast, FPN is under-expressed in several models of PD including MPTP and 6-Hydroxydopamine (6-OHDA) [38, 39]. This decrease in FPN may be caused by impaired expression or membrane trafficking of APP and ceruloplasmin in PD and relevant models [40–43].

More recently, with the advance of magnetic resonance imaging, a strong correlation between disease severity and levels of iron in the SNc has been identified [44, 45]. To a lesser extent, iron overload also seems to precede atrophy in the striatum [45]. Despite these early stage iron changes with disease and the potential for its use as a biomarker for disease progression, it still remains to be determined whether iron is causal or a downstream effect of PD.

## Role of α-synuclein in synaptic maintenance

Aggregated α-syn is a central neuropathological feature in PD patients and mutations in the *SNCA* gene encoding α-syn result in familial PD [46, 47]. Neuronal expression levels of α-syn are heterogenous throughout the brain. The high expression in SNc, caudate nucleus, putamen and ventral pallidum closely tracks the dopaminergic regions affected in PD [48]. The physiological role of α-syn is poorly understood, however it is implicated in various cellular processes. A location within presynaptic terminals as well as a neuroprotective capacity on nerve terminal injury and SNARE (soluble *N*-ethylmaleimide-sensitive-factor attachment protein receptor) complex disruption [49, 50] consolidate a theory that α-syn has a role in neurotransmitter storage and release within the synapse [51]. Spatial and working memory deficits upon α-syn deletion in a mouse model support the requirement of this protein to maintain synaptic function [52, 53]. The function of α-syn within presynaptic vesicles may be as a molecular chaperone in folding SNARE proteins [49]. This is similar to the homologous 14-3-3 protein [54] and cysteine-string protein α (CSPα) [55]. Expression of 14-3-3 protein is increased upon α-syn depletion [52], whilst neurodegenerative depletion of the latter can be rescued by overexpression of α-syn [49]. It is now understood that the fusion and clustering of SNARE-associated vesicles to the synaptic plasma membrane can be regulated by α-syn association with vesicle-associated membrane protein 2 (VAMP2/synaptobrevin-2) [50]. By keeping VAMP2 in close proximity with the t-SNAREs, α-syn can control stimulated neurotransmitter release through a role in vesicle clustering.

α-Syn has also been suggested to modulate vesicle size and the releasing properties of synaptic vesicle recycling and reserve pooling [51, 56]. An argument for α-syn involvement in overall modulation of DA recruitment and homeostasis arises from known interactions with VMAT2 during vesicle filling as well as with DAT required for DA reuptake [57, 58].

Cellular evidence indicates α-syn is able to alter iron homeostasis, aligning with an IRE being discovered within its 5′-promotor region [59]. Endocytic/exocytic trafficking is a fundamental component of iron homeostasis and as recently reported, both α-syn and TfR colocalise on the membrane surface. Depletion of α-syn results in an accumulation of Tf/TfR complex within the endosome [60]. Dynamin 1, as an additional target for CSPα complexes, is another α-syn interactor [61]. This provides new insights into a more general role for α-syn in molecular chaperoning of early clathrin-mediated endocytosis. The ability of α-syn to control clathrin-mediated endocytosis therefore suggests that this protein may be another regulator of the intracellular iron pool and thus requires further investigation.

Of note, α-syn may also play a role in DA synthesis through binding to inhibit TH; the rate limiting iron-dependent enzyme for DA biosynthesis [62]. More generally, α-syn also regulates monoamine transporters [63] and interacts with the signalling protein ARPP16/19; a DA- and cAMP-regulated neuronal phosphoprotein family member involved in regulation of DA signalling pathways [64]. Since the primary location of α-syn is in the synapse it is likely that any modification to the protein will have detrimental effects on synaptic transmission and pathogenesis in α-synucleinopathies such as PD.

## Post-translational modifications to α-synuclein on the membrane

α-Syn undergoes several post-translational modifications (PTMs) including acetylation, phosphorylation, oxidation, nitration, ubiquitination and truncation. These PTMs regulate α-syn structure and physiological function. They could also be linked to the aggregation and/or oligomer formation of α-syn as all have been found extensively in LBs. This review will focus on N-terminal acetylation; responsible for the constitutive structure of α-syn found in vivo*,* as well as phosphorylation and oxidation; the most common PTMs found in LBs and associated with OS.

In its physiological state, α-syn is constitutively N-terminally acetylated [65, 66]. This region is particularly rich in lysines that are known to be involved in the formation of an α-helical structure upon lipid interaction [67]. Acetylation of lysines is a reversible reaction that impacts on multiple cellular pathways. The acetylation of lysines 6, 34, 45 and 96 on α-syn have all been reported in the brain [68] and these acetylated forms have been purified from LBs [69]. Modification of these same residues by aldehydes (e.g. products of DA catabolism or lipid peroxidation) may affect α-syn’s membrane binding capability via acetylation [67]. Of relevance to our proposed concept are effects of N-terminal acetylation, charge and curvature of vesicles on α-syn binding. The binding of α-syn to lipid vesicles with high negative charge content is essentially unaffected by N-terminal acetylation irrespective of curvature. However, binding to vesicles containing lower negative charge is increased, with stronger binding observed for vesicles with higher curvature; properties that relate closely to synaptic vesicles [70, 71]. This is supported by N-terminal acetylation of α-syn being shown, by nuclear magnetic resonance spectroscopy (NMR), to produce more pronounced binding to membranes of increased curvature and moderate charge [70]. Of note, a later study summised that N-terminal acetylation only affected the ‘weak’ binding of α-syn to zwitterionic vesicles with no change in phospholipid membrane binding affinity [72]. This has led to the speculation that if N-terminal acetylation is involved in lipid binding it may be mediated through other binding partners.

Several phosphorylation sites have been identified on α-syn either at tyrosine, threonine or serine (e.g. Y39, S87, Y125, and S129). However, S129 is the locus most typically affiliated with PD as this site is phosphorylated in around 90% of α-syn deposits in PD patients compared to 4% in controls [73]. Information from site directed mutation studies have mostly concentrated on mechanisms of toxicity related to aggregation (see below) but its role in membrane interaction is being elucidated. Collectively, data on the phosphorylation site at S129, S87 and Y39 support a concept that this PTM results in an inability of α-syn to bind membranes [74–78]. Of relevance to DA trafficking, phosphorylation of membrane associated α-syn at S129 alters neurotransmitter uptake [79]. It is yet to be confirmed whether a similar effect occurs in synaptic vesicle formation associated with VAMP2. An attempt to address how α-syn phosphorylation impacts upon interactions with other proteins has led to the discovery that phosphorylation of α-syn at S129 promotes Rab8a binding and mediates toxicity. Rab8a is a small guanine nucleotide binding protein implicated in coordinating vesicle trafficking [80]. Similar to phosphorylation, the methionine oxidation of α-syn leads to a decreased affinity for biological membranes [81]. In vitro reports have identified this modification to alter the α-syn aggregation pathway, but its effect within the synapse remains to be fully characterised. This is partly due to its transient nature under physiological conditions. While methionine oxidation of α-syn impairs degradation through the 20S proteasome [82], in-cell NMR now indicates that oxidative modification to methionine residues at the N-terminal region of α-syn is reversible as it can be enzymatically repaired [83].

## Structure changes to α-synuclein that induce oligomerisation

Recent studies using more physiological conditions in the purification of α-syn have suggested an equilibrium between the tetrameric α-helical and monomeric forms that coincides with N-terminal acetylation [65, 66]. Disruption of the α-helical tetramer may trigger the formation of alternative soluble oligomeric structures as intermediaries of the insoluble fibrils observed in LBs [65]. Indeed intracellular cross-linking experiments have indicated that the presence of this tetramer can be disrupted by familial PD point mutations in α-syn [84]. This theory still remains to be confirmed as other groups report an intrinsically disordered structure when the protein is purified under similar conditions [85]. In-cell NMR has also shown that α-syn retains an intrinsically disordered state, rather than an assembled tetrameric α-helical structure, when the protein is introduced to the cell by electroporation [86].

As with many other amyloidogenic neurodegenerative diseases, evidence suggests that it is the soluble oligomeric forms of α-syn preceding fibril formation that mediate pathogenesis [87]. Disruption of the intracellular tetramer may be the first step in this pathway [84]. Modification to the conformation of the monomer or its oligomeric state is likely to be highly susceptible to genetic alteration, PTMs and interaction with ligands such as transition metals or DA [88–90]. While it is important to identify how an increase in the propensity for aggregation can occur, it is unlikely that only a single modification to α-syn is responsible for PD pathology within the SNc. Indeed, an equilibrium of α-syn species is likely to exist within the SNc at any one time and the function of α-syn or its pathological effect will rely on a shift in this equilibrium.

### Point mutations in α-synuclein associated with pathology

Mutations in α-syn including A30P, A53T, E46K and more recently identified H50Q and G51D, result in early onset PD. Li et al. [91] show that although the monomeric structure of wild-type (WT), A30P and A53T is similar, mutated forms aggregate in vitro at a faster rate. H50Q decreases the solubility of α-syn by up to 10-fold [92] whereas G51D may promote an alternative mechanism of pathogenesis as aggregation properties are reduced [93, 94]. Such a mechanism may be associated with the chaperone like properties of α-syn as G51D reduces lipid-binding function [95]. Enhanced vulnerability to mitochondrial impairment and oxidative stress have also been suggested as potential modes of action for this variant [93]. Whilst A53T shows an increased rate of fibril formation [96], A30P is more likely to aggregate into the protofibril oligomeric species while not progressing to the full fibril forms. In contrast to the other familial PD mutations in α-syn, E46K does show structural changes of the monomeric species that modify the polarity of the amphipathic repeat region [97, 98]. The E46K aggregates more rapidly into fibrils [99, 100] but these fibrils are morphologically similar and protofibrils are fewer than with WT α-syn [101]. Intriguingly, Mbefo and colleagues [102] identify that E46K increases phosphorylation at S129 and alters the subcellular localisation of α-syn, suggesting that this may cause enhanced aggregation.

Currently, all the familial PD mutations in α-syn increase the propensity of the protein to aggregate and therefore suggest this to be a key component of the disease associated neurotoxicity. However whilst H50Q, A53T and E46K appear to promote fibril formation, A30P has a greater prevalence in stabilising the oligomer species. It remains to be identified whether there is a defined species of aggregated α-syn that is the toxic species or, as is more likely, a range of forms are responsible for neuronal death in the SNc.

### Modification of α-synuclein by oxidation

Oxidation of the 4 methionine residues located in the N-terminal (M1 and 5) and the C-terminal (M116 and 127) of α-syn produce methionine sulfoxides that inhibit fibrillisation. The degree of this inhibition is proportional to the number of oxidised methionine residues [103]. In addition, oxidative modifications to the tyrosines via nitration induce a partial folded conformation that stabilises soluble oligomers and stops elongation into fibrils. These oligomeric species are thus formed along aggregation pathways distinct from ones used in amyloid fibril formation [104–106]. In the presence of H_2_O_2_ all 4 methionines are converted to sulfoxides [107] and rotenone (used as a neurotoxic Parkinsonian model) results in methionine oxidation and subsequent intracellular aggregates [108].

### Modification of α-synuclein by phosphorylation

The phosphorylation status of α-syn has a marked influence on aggregation and toxicity. However, it remains to be confirmed whether phosphorylation promotes or prevents aggregation and toxicity (e.g. [109]). Disparities may arise from there being no established aggregated form of α-syn that is predominantly toxic [110–114] and the different kinase efficiencies for phosphorylating α-syn. In vitro biochemical studies with phosphorylation of α-syn at S129 by casein kinase (CK) 2 results in greater fibril formation than with unphosphorylated α-syn [73], conversely polo-like kinase (PLK) 2 phosphorylation has comparable fibrillisation kinetics to the WT protein [115]. The subcellular location of α-syn also plays a role in which kinase phosphorylates the protein. CK2 and G protein-coupled receptor kinase (GRK) 3, 5 & 6 contribute to S129 phosphorylation of membrane-associated α-syn, whereas cytosolic α-syn is phosphorylated exclusively by CK2 [79]. Most cell culture studies associate S129 phosphorylation with increased formation of soluble oligomers [116, 117], cytoplasmic and nuclei aggregates [116, 118], and cytoplasmic inclusions [119, 120]. In contrast the results from multicellular animal models are less clear with phosphorylated S129 promoting [73, 116–121], preventing or having no effect on inclusion formation [75, 109, 122–128]. Discrepancies in these studies originate from a reliance on α-syn phosphomimetic mutations that do not fully recapitulate the real phosphorylation states of α-syn [115, 123].

The presence or absence of additional factors are likely to be a feature of the variances in phosphorylated S129 α-syn aggregation. These may largely derive from the buffer conditions in which the samples are prepared. In addition, C-terminal methionine sulfoxides impair Y125 phosphorylation by the major tyrosine kinase Fyn [83]. As phosphorylated Y125 primes the efficient modification of S129 by CK [129], reduction in Y125 phosphorylation is likely to also diminish modifications of S129. This would support the presence of an age- and disease-dependent decline in α-syn phosphorylation in models and patients of PD [127].

### Modification of α-synuclein by iron

Despite Fe^2+^ being the predominant form within the cell, Fe^3+^ has the greater affinity for α-syn (Fe^2+^; 5.8 × 10^3^ M^−1^, Fe^3+^; 1.2 × 10^13^ M^−1^) at D121, N122, and E123 [88, 130]. This suggests that within an aerobic environment the intracellular Fe^2+^ could have a greater affinity upon oxidation to Fe^3+^. The resulting H_2_O_2_ and hydroxyl radicals byproducts could augment OS [131] and in turn lead to the oxidation of α-syn at methionines, as observed with other reduced metals and oxidised lipids [81, 132].

The affinity between α-syn and divalent metals such as Fe^2+^ is also altered by PTMs such as phosphorylation. Peptide studies show that phosphorylation at S129 or Y125 increase the binding affinity for Fe^2+^ but not Fe^3+^ at residues 107–140, thereby altering the residues involved in the binding site [133]. Despite confirmation being required in the full-length protein, this suggests that phosphorylation may increase the available pool of iron that promotes intracellular α-syn aggregation.

Binding of Fe^3+^ directly, or as a result or Fe^2+^ oxidation, alters the morphology of α-syn fibrils and accelerates aggregation in both WT and mutant variants including E46K [134]. In the presence of unilamellar vesicles, the addition of Fe^3+^ to α-syn results in the formation of stable oligomers that impair membrane conductance and lead to neurotoxicity [135].

### Modification of α-synuclein by dopamine

Several in vitro studies illustrate that DA can modulate the aggregation of α-syn to form oligomers not considered direct intermediates on a pathway to amyloid fibril formation [89, 90]. DA modification of α-syn is through the oxidation of all 4 methionines. Substitution of these residues significantly reduces the propensity of α-syn to form kinetically stable oligomers [136]. Despite fibrils being formed after extended incubation in the presence of DA, these are less stable and susceptible to fragmentation [137]. The oxidative intermediates of DA also have the ability to bind and induce α-syn aggregation. Aminochrome promotes the formation and stabilisation of neurotoxic protofibrils of α-syn [90, 111] and leads to the formation of adducts (e.g. 5,6-indolequinone) that subsequently also bind α-syn [138, 139].

Despite an ability of Fe^2+^ to auto-oxidise in an aerobic environment, this Fenton reaction is greatly assisted in the presence of DA, to produce hydroxyl radicals. In the presence of DA, the ability of Fe^3+^ to promote α-syn fibril formation is completely inhibited [89, 140] and identifies DA as a key modulator of α-syn oligomer formation. The relationship between α-syn and iron has also been studied in vivo*.* Overexpression of various α-syn mutants in the presence of iron and DA or H_2_O_2_ induce the formation of aggregated α-syn [141].

## Posttranslational modifications to α-synuclein regulate dopamine and iron transport

It is possible that preferential vulnerability to PD-related neurotoxicity in a subgroup of SNc dopaminergic neurons arises from the convergence of different cellular risk factors. It is becoming increasingly evident that α-syn has physiological roles in both iron and DA homeostasis. More established in the literature is the hypothesis that α-syn can regulate DA through synaptic vesicle docking and fusion, recycling of vesicles and import through the DAT/VMAT2 receptors [57, 58]. However, α-syn can also mediate the production of cellular DA through the regulation of iron that is required for TH activity [118]. This regulatory function may also be through a role α-syn has in receptor mediated endocytic trafficking. Through an interaction with dynamin [61], α-syn is proposed to modulate clathrin-coated endocytosis, of which the best characterised model is TfR mediated iron import. In support of this being a pathway in which α-syn may regulate cellular iron levels, it has recently been identified that α-syn ablation alters the level of TfR and iron within neurons [60].

**Fig. 1:**
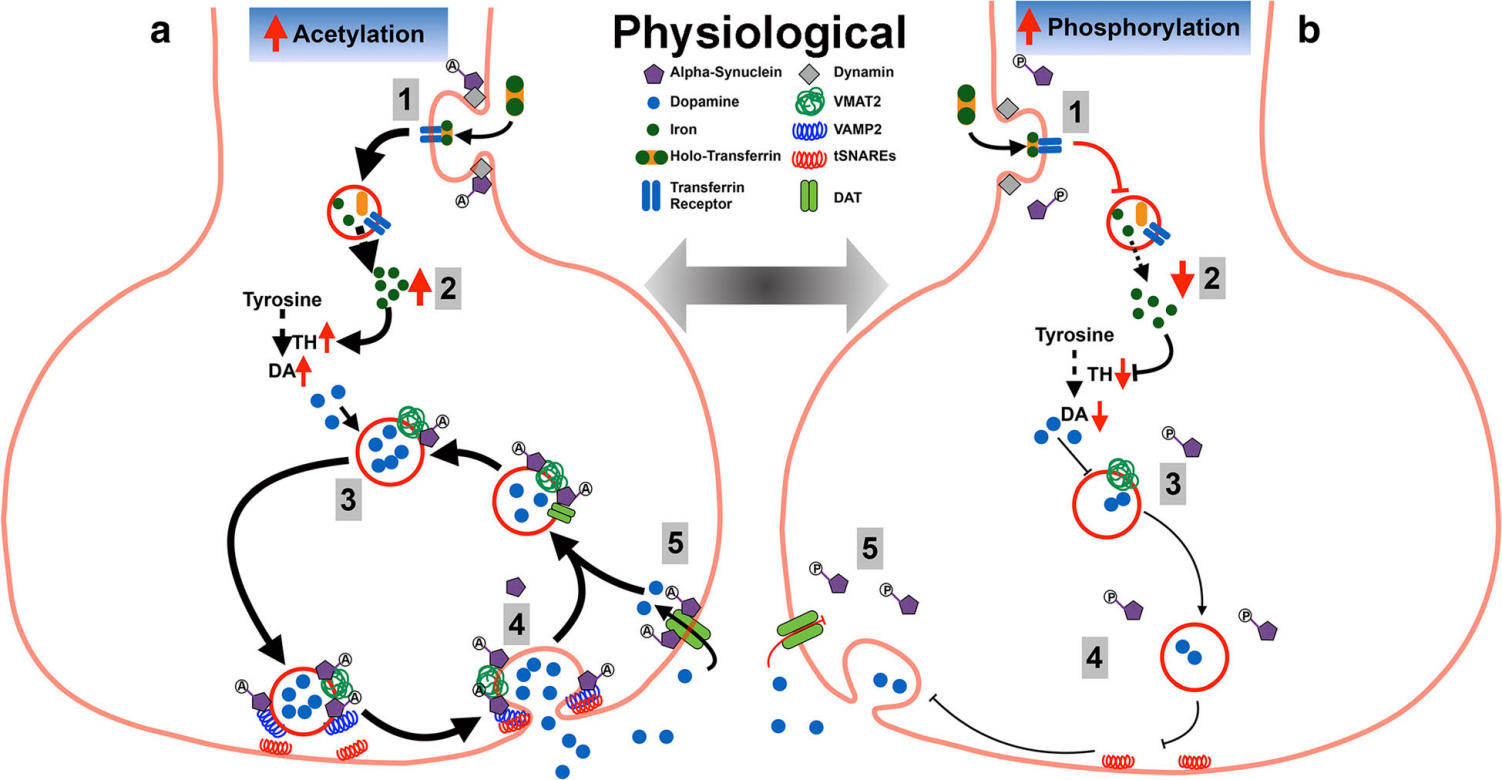
A working model that illustrates the functional role of post translationally modified α-synuclein in normal physiology. **a** N-terminal acetylation of α-syn facilitates dynamin-mediated endocytosis of TfR and internalisation of iron (1). An appropriate intracellular iron level is tightly controlled to maintain neuronal function including DA production by the iron-dependent enzyme TH (2). DA is incorporated into synaptic storage vesicles through α-syn binding to VMAT2 (3). Upon stimuli, VAMP2 then binds to the t-SNARE protein to allow fusion with the synaptic membrane and release of DA into the cleft (4). Recycling of DA back into the pre-synaptic neuron through the DAT receptors also requires binding to α-syn on the membrane and subsequent reinternalisation into synaptic vesicles through the α-syn/VMAT2 complex (5). **b** When iron or DA transport is required to be reduced in physiological conditions, α-syn is phosphorylated or oxidised (not shown) to decrease lipid affinity. A lack of membrane bound α-syn reduces neuronal iron import through TfR endocytosis (1), production of DA by TH(2), DA incorporation into synaptic vesicles (3), reduced DA release into the synaptic cleft (4) and/or DA recycling within the presynaptic neuron (5)

Despite an increased comprehension of α-syn function, there are still conflicting reports on whether α-syn promotes or inhibits vesicle trafficking (e.g. [51, 56]). We propose that this confusion is largely caused by the perception that the regulation of functional α-syn is only at a translational level. Similar to other cell modulatory pathways (e.g. the cell signalling transduction network), it is feasible that PTMs to α-syn control vesicle trafficking. Indeed, phosphorylation and oxidation of α-syn are detrimental to α-syn binding to lipids [74–77, 81] whereas acetylation of α-syn induces a greater affinity [70]. This suggests that phosphorylated or oxidised forms of α-syn have an increased propensity to be retained within the cytoplasm whereas native N-terminal acetylation of α-syn permits attachment to vesicles membranes either in its own right or through interaction with partner proteins. The pool of cytoplasmic iron within neurons is tightly monitored and if levels become too low as to impair the activity of enzymes such as TH, then overriding regulatory pathways are implemented. In the current working hypothesis on α-syn function in iron and DA trafficking (Fig. 1a) we reinforce a theory that α-syn binding to lipid membranes requires N-terminal acetylation. Parallel with promoted translation of α-syn when iron is required by the cell (due to the IRE within the 5′ promoter region), lipid binding of the N-terminal acetylated form facilitates the dynamin-mediated endosomal trafficking of TfR and controls iron internalisation. In parallel, cellular DA production through TH activity requires the storage of this oxidant in synaptic vesicles to minimise cytosolic degradation and free radical production. The incorporation of DA into synaptic storage vesicles is facilitated through α-syn binding to VMAT2. Upon stimuli, VAMP2 then binds to its corresponding t-SNARE protein to allow fusion with the synaptic membrane and release of DA into the cleft. Recycled DA, not required by the post-synaptic neuron, is then imported back into the pre-synaptic neuron through the DAT receptors that also bind α-syn on the membrane. DA is internalised into synaptic vesicles through the α-syn/VMAT2 complex.

In physiological conditions when either iron and/or DA transport requires to be altered, we propose that this can be mediated through post translational modification of α-syn. Phosphorylation, or conditions that give rise to the oxidation of α-syn, reduce the α-syn binding capacity to lipid membranes, thereby no longer promoting endocytosis or vesicle trafficking (Fig. 1b). TfR controlled iron import into the cell will be decreased, in turn decreasing TH activity and lowering DA production [118]. Phosphorylation will similarly reduce the ability for α-syn to bind membrane DAT/VMAT2 or VAMP2 and thus reduce DA incorporation into synaptic vesicles or recycling within the presynaptic neuron. This concept on the physiological function of α-syn in iron and DA homeostasis describes how modifications to the protein individually affect iron and DA homeostasis. However, it is highly probable that other PTMs are involved and that the total α-syn population present in a cell is made up of multiple PTMs dependent on the cell’s specific requirement in a certain location. Therefore, it is feasible that phosphorylated α-syn may be implemented in one area of the neuron to reduce iron import, while in the synapse it may be alternatively modified (i.e. N-terminally acetylated) to increase DA recycling and trafficking.

## Posttranslational modifications to α-synuclein increase vulnerability to neurotoxicity when dysregulated

**Fig. 2:**
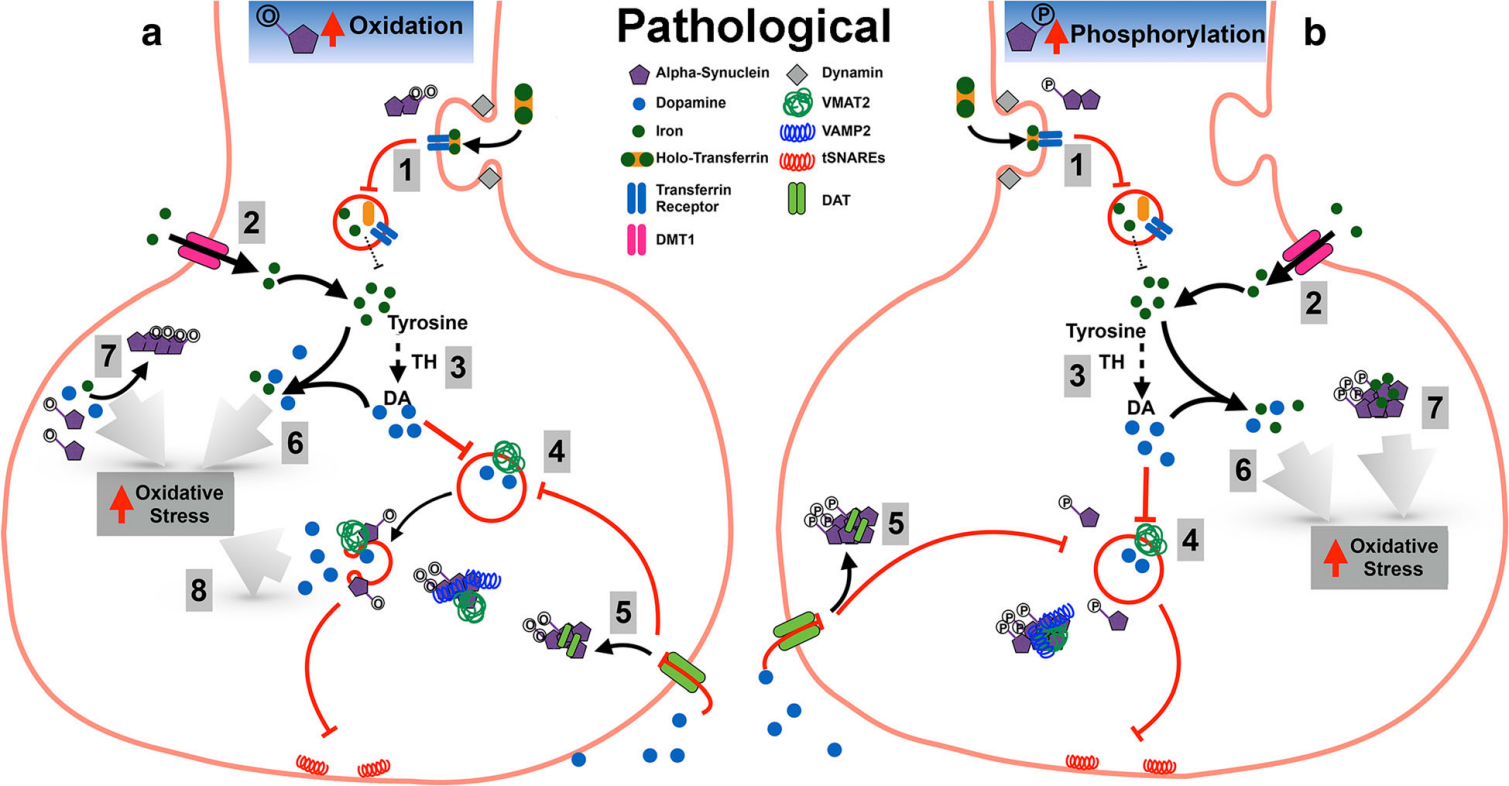
A schematic on how unregulated phosphorylation or oxidation of α-synuclein can disrupt iron and dopamine trafficking to lead to increased oxidative stress. Increased oxidation (**a**) or hyper-phosphorylation (**b**) of α-syn strongly reduces iron import through endocytosis of TfR (1). This leads to an initiation of alternative compensatory import mechanisms such as DMT1 expression to maintain cellular function (2). Elevation of iron by DMT1 restores DA production (3) but a lack of membrane bound α-syn causes impaired VMAT2-assisted transfer of DA into synaptic vesicles (4). The cytoplasmic location of oxidised (**a**) or phosphorylated (**b**) α-syn will also alter the location of DAT receptors on the cell surface and reduce recycling of extracellular DA (5). Elevation in cytoplasmic DA within a high labile iron environment generates toxic DA reactive quinones and promotes oxidative stress (6). Increased cytoplasmic DA may also lead to further post-translational modifications of α-syn, specifically the oxidation of methionines, thus increasing a propensity for α-syn to form aggregated species (7) and disrupt lipid membranes via lipid peroxidation (8). Once the oxidative damage produced from the interplay between modified α-syn, iron and DA outweighs protective antioxidant mechanisms, neuronal damage will be cyclically accelerated

The pathological relevance of α-syn in these pathways is when PTMs become dysregulated and accumulate to detrimental levels. Upon hyper-phosphorylation or excessive oxidation of α-syn, TfR endocytosis is likely to be impaired (Fig. 2). The subsequent reduction in intracellular iron could lead to alternative compensatory import mechanisms being initiated in order to maintain cellular function (e.g. DMT1 [36, 37]). An attempt by SNc neurons to rescue metabolic function by increasing DMT1 expression, as observed in PD [37], may have further detrimental consequences in iron-mediated OS susceptibility. Furthermore, DA produced through restored TH activity will not be correctly incorporated into synaptic vesicles via VMAT2 due to the absence of membrane bound α-syn. Elevated cytoplasmic DA within a high labile iron environment is likely to consequentially generate toxic DA reactive quinones along with reactive species that promote oxidative stress and mitochondria dysfunction. Along with impaired neurotransmitter uptake, conditions of high phosphorylation or oxidation would deplete synaptic DA stores and compound neuron dysfunction during synaptic transmission. In addition PTM induced changes to the aggregation of α-syn may compound toxicity through consequential modifications to DA, iron and related auto-oxidation species [89, 90, 103, 134]. As confirmed by the prevalence of phosphorylated α-syn in LBs, this PTM accelerates aggregation [73]. Whilst this can be further exacerbated by the presence of iron, the addition of an oxidant such as DA, steers a profile more towards maintenance of oligomeric species and protofibrils [89, 140]. Oxidation alone also has this affect on α-syn and these soluble oligomeric and protofibrils species may be more detrimental to the cell through an increased capacity to generate reactive oxygen and nitrogen species. The presence of soluble oligomeric species of oxidised α-syn within close proximity to synaptic vesicle membrane may also lead to ROS-induced lipid peroxidation and subsequent release of DA incorporated through the already impaired VMAT2 mechanism. An iron and lipid dependent form of cell death called ferroptosis has recently been identified as a major feature in models of PD [142]. The oxidation of α-syn may be a key component of this pathway that requires further investigation.

## Concluding remarks

Experimental elucidation of DA and iron metabolism in neurons have slowly pointed to α-syn as a key regulator in synaptic and endosomal vesicle trafficking with links to PD pathology. Understanding the complexities of PTMs to α-syn has also increased during the same period. However relevance of these changes to iron and DA dyshomeostasis in sporadic PD have yet to be determined. The relationships these have to genetic polymorphisms at the *SNCA* locus associated with PD also remain elusive. Overall, reviewed information concurs with our proposed hypothesis that α-syn is intimately linked with iron and DA physiology. The invoked dysregulation to either pathway present in PD and a range of other synucleinopathies (e.g. DLB and MSA), is therefore suggested to be directly linked to neuropathology in cell populations that are vulnerable in these diseases. There is therefore a fundamental necessity for continued research into understanding how the modifications to α-syn described here and by others, adapt the protein’s normal role within the neuron and in synucleinopathies. It is the hope that risk genes identified in genome-wide association studies will assist in the identification of not only pathological pathways in PD but also provide the necessary assistance in solving the physiological pathways related to α-syn.

While this review has provided a proof of concept to only part of the complexity in the disease, it highlights that the pathology does not originate from a single process. A complex combination of genetic and environmental factors will lead to pathology in most PD forms and it is conceivable that the pathways involved in alterations to iron and DA transport may vary from one individual to another. Consequently, therapeutic strategies for preventing or slowing down disease progression are likely to require personalisation for each PD case. As such, greater characterisation and validation of biomarkers used in disease diagnosis will substantially assist in identifying the disease subpopulation in which a patient falls into. It will also enable them to be treated at an earlier disease time point with a drug that they will have greater response to.

## Abbreviations

α-syn: α-synuclein
6-OHDA: 6-hydroxydopamine
APP: Amyloid-β precursor protein
CK: Casein kinase
CSPα: Cysteine-string protein α
DA: Dopamine
DAT: Dopamine transporter
DMT1: Divalent metal transporter 1
DOPAC: Dihydroxyphenylacetic acid
Fe^2+^: Ferrous iron
Fe^3+^: Ferric iron
FPN: Ferroportin
H_2_O_2_: Hydrogen peroxide
IRE: Iron response element
LBs: Lewy bodies
LN: Lewy neurites
MAO: Monoamine oxidase
MPTP: 1-methyl-4-phenyl-1,2,3,6-tetrahydropyridine
NAC: Non-amyloid component region
OS: Oxidative stress
PD: Parkinson’s disease
PLK: Polo-like kinase
PTM: Post-translational modification
ROS: Reactive oxygen species
SNARE: Soluble *N*-ethylmaleimide-sensitive-factor attachment protein receptor
SNc: Substantia nigra pars compacta
SNr: Substantia nigra pars reticulata
TfR: Transferrin receptor
TH: Tyrosine hydroxylase
VAMP2/synaptobrevin-2: Vesicle-associated membrane protein 2
VMAT2: Vesicular monoamine transporter 2
VTA: Ventral tegmental area
WT: Wild-type
